# Preparative Purification of Recombinant Proteins: Current Status and Future Trends

**DOI:** 10.1155/2013/312709

**Published:** 2013-12-17

**Authors:** Mayank Saraswat, Luca Musante, Alessandra Ravidá, Brian Shortt, Barry Byrne, Harry Holthofer

**Affiliations:** Centre for Bioanalytical Sciences (CBAS), Dublin City University (DCU), Dublin 9, Ireland

## Abstract

Advances in fermentation technologies have resulted in the production of increased yields of proteins of economic, biopharmaceutical, and medicinal importance. Consequently, there is an absolute requirement for the development of rapid, cost-effective methodologies which facilitate the purification of such products in the absence of contaminants, such as superfluous proteins and endotoxins. Here, we provide a comprehensive overview of a selection of key purification methodologies currently being applied in both academic and industrial settings and discuss how innovative and effective protocols such as aqueous two-phase partitioning, membrane chromatography, and high-performance tangential flow filtration may be applied independently of or in conjunction with more traditional protocols for downstream processing applications.

## 1. Introduction

There is an ever-increasing requirement for protein production in industrial and academic settings for a variety of applications. These include exploratory research, drug discovery initiatives, biopharmaceutical production, target validation, and high-throughput screening. Some 200 recombinant proteins-based biopharmaceuticals have gained approval so far for human therapeutic and/or diagnostic use and in excess of 350 are currently in late-stage clinical trials [1]. Notably, pharmaceutical research and manufacturers of America (http://www.phrma.com/) have estimated that its member companies are developing or providing funding for pursuing the development of some 320 biotechnology medicines, with most of these being protein-based. For example, thirty therapeutic complete monoclonal antibodies and three antibody fragments have been approved so far by US FDA as of January 2012 and over 240 are in the developmental stages [1–3]. In addition to biopharmaceuticals, structural genomic initiatives also require milligram (mg) amounts of protein for three-dimensional (3D) structure representations. According to TargetDB statistics as of the first of March 22, 2012, some 295,015 targets have been deposited, out of which 202,005 have been cloned with 128,852 being expressed and 47,784 subsequently purified (http://targetdb-dev.rutgers.edu/TargetDB-dev/stats.html). While currently employed large-scale production strategies yield cell culture/fermentation titres containing up to tens of grams per litre, there is a subsequent need to ensure that all impurities are removed and that sufficient amounts of highly purified protein are obtained for the desired application (http://www.genengnews.com/gen-articles/downstream-bottlenecks-more-than-just-perception/4129/). Furthermore, escalating demands for increased protein titres, primarily for economic reasons, have shifted the bottleneck step from production to purification, with downstream processes (inclusive of purification) representing between 45 and 92% of the total cost of manufacturing a recombinant protein [4, 5]. Hence, devising an efficient and economical purification strategy is a key challenge and one which is faced by industrial and, to a lesser extent, by academic laboratories. In this review, we provide an overview of the traditional and more recently developed protein purification strategies currently being employed for industrial and academic applications, with particular emphasis on methodologies implemented for the production of recombinant proteins of biopharmaceutical importance.

## 2. Chromatography Material Functionalities

### 2.1. Affinity Chromatography

There are numerous ways in which an affinity-based method may be employed for the purification of recombinant proteins. The most common example of an affinity process is protein-A chromatography, which has been applied for over a decade in industrial and academic settings for the capture and purification of antibodies (immunoglobulins) [23]. In spite of several notable drawbacks associated with the use of this technology, primarily the ability of protein-A to leak into the mobile phase and the high associated costs, it is still widely used as a capture step in large-scale purification of monoclonal antibodies (mAbs), including those of therapeutic use [24]. Recombinant protein-A-bound resins with high binding capacities (e.g., MAbSelect Sure by GE Healthcare) are commercially available and can withstand the harsh sterilising conditions introduced between cycles of industrial downstream processing. What makes protein-A-based applications more lucrative for industrial uses, in spite of their obvious disadvantages, is their ability to indirectly remove viruses (such as SV40, X-MuLV, and MMV) from the feed [25]. Moreover, the ability to capture mAbs directly from clarified harvest without any pretreatment and very high selectivity leading to removal of most host cell proteins are two distinct advantages of protein-A chromatography. Another affinity-based strategy which has been utilised for the easy purification of recombinant proteins is the use of fusion tags, namely amino acid sequences which are attached to recombinant proteins and have selective and highaffinities for a chemical or biological ligand which is immobilised on a chromatography column, hence permitting purification of the recombinant protein. Commonly used affinity tags (and their cognate binders) which permit purification of a selection of tagged proteins are outlined in Table 1.

In particular, the histidine (His) tag has been frequently used to facilitate purification of recombinant proteins from bioprocess mixtures [26–30]. This tag is comprised of a sequence of six or more histidine residues which are added to either the N or C terminal of the recombinant protein of interest and exhibits a high affinity towards metal ions such as nickel (Ni) and zinc (Zn). Immobilised metal affinity chromatography (IMAC) uses a matrix which has a metal-chelating group available for metal binding, and the remaining coordination sites on the metal bind His residues attached to the recombinant protein, thus enabling its purification via elution with a metal chelator such as imidazole. IMAC is commonly used as a protein purification strategy in academic [31, 32] and industrial settings [33]. Furthermore, several therapeutic candidate protein pharmaceuticals purified using IMAC are currently in clinical studies [34–38]. Naturally occurring metal-binding proteins and the presence of histidine and cysteine-rich spots in superfluous proteins compete with tagged protein to bind to the column and interfere with IMAC often resulting in contamination of the final product. However, it is a common initial purification method for HIS tagged recombinant proteins and purities up to 95% with recovery of 90% can be achieved in a single step [39]. Moreover, IMAC resin is unaffected by protease activity in the feed unlike many other biological affinity procedures. HIS tagged proteins can be eluted from IMAC by mild elution conditions which would help in recombinant proteins retaining their activity and/or native folding. However, possibility of heavy metal leaching from the column during purification can be of concern [40] and testing for it would require additional costs for validation. Finally, the removal of HIS tag would require expensive proteases and additional chromatographic steps needed to purify the target protein would further increase the costs.

Alternative tags (e.g., FLAG, Softag1, and Softag3, Table 1) bind to specific mAbs raised against them (such as ANTI-FLAG M1 Agarose Affinity Gel from Sigma) which, in turn, can be used for immunopurification of tagged proteins. However, there is an increased cost associated with the use of mAbs, while harsh chromatography conditions may have a deleterious effect on the ability of the antibody to bind to its cognate antigen over multiple purification cycles. Strep tag II is an octapeptide (WSHPQFEK) which recognises streptavidin and can be eluted from the streptavidin-containing column using biotin analogues. Hence, this tag can be used for purifying proteins expressed in a selection of different host systems [10, 41]. Notably, these tags can be removed via proteolytic cleavage of the linker region between the ligand and analyte, and passing the analyte through the tag-binding column for a second time can yield pure protein free of nonspecific binders and proteases. However, as mentioned above, these proteases are expensive and the use of these tags would increase the cost of downstream processing.

Downstream processing of biopharmaceuticals usually represents the major cost of protein manufacturing, and a single-step isolation of a recombinant protein using an affinity ligand with high selectivity and stability is therefore an ideal strategy [3]. Affinity chromatography has been used for purification of proteins for many years, and recent progress in combinatorial and *de-novo* ligand design seem very promising [42]. The design of ligands which can be used for purification of target protein(s) can be performed in various ways, including protein structure-based design, function-based design, and through combinatorial approaches such as bacteriophage display, ribosome display, and systematic evolution of ligands by exponential enrichment (SELEX) [42]. Structure-based design of ligands takes advantage of the known 3D structure of the target protein and identifies a site for ligand binding which has a known active site [43], a surface-solvent exposed area [44] or a site used by a natural ligand to bind [45]. The designed ligand is docked to the protein structure and binding mode, affinity, and molecular parameters are calculated using different algorithm-based software. These parameters can also be estimated experimentally using, for example, Surface Plasmon Resonance (SPR) analytical platforms (e.g., Biacore from GE Healthcare or Octet by ForteBio), which have dedicated software for data analysis, quartz-crystal microbalance (QCM) analysis, or isothermal titration calorimetry. There are reports of successful ligand design approaches in the literature. As an example, a biomimetic dye-ligand designed for L-lactate dehydrogenase (LDH) was used for its purification from a selection of different sources, including bovine heart, chicken liver, pig muscle, and pea seeds [46]. Other examples of successful dye-ligand-based purification approaches have been reported [47–49]. This illustrates the potential and selectivity of using this approach for purification from complex sample matrices. 

Function-based design is implemented when the structure of the target protein is unknown. Here, the purification strategy selected for use is based on any functional information available for the target protein. One example is Cibracon blue 3GA, which adopts nucleotide-like conformations and can be used for the purification of nucleotide-binding proteins [50]. Biomimetic compounds mimicking known substrates, inhibitors, or cofactors of target proteins can also be used as affinity ligands. Some of the dye adsorbents for protein purification are commercially available from various companies.

Synthetic peptide or phage/ribosome display libraries have been used to identify peptide ligands which bind to target proteins with high affinities. Based on these approaches, a hexapeptide (FLLVPL) was identified from a synthetic peptide library and used for the purification of human fibrinogen [51], while other high-affinity ligands for target proteins can be identified from phage libraries through a process termed biopanning [52]. For example, Benlysta was approved by FDA, in 2011, for the use in lupus and the antibody was discovered by phage display technology and further developed for commercial use. SELEX was used to identify a deoxyribonucleic acid (DNA) aptamer which was able to purify human L-selectin-immunoglobulin fusion protein expressed in Chinese hamster ovary (CHO) cells [53]. More recently, a small molecule affinity ligand for human growth hormone (hGH) was identified through screening of a combinatorial library which was enriched through a combination of computational and online screening. Using this ligand, hGH was purified from microfiltered *Escherichia coli *(*E. coli*) lysate with high levels of recovery (83%) and purity (80%) observed [54]. Another important aspect of this study is that small molecule affinity ligands are more advantageous than biomolecule ligands as they can tolerate harsh conditions such as in the routine sterilisation of resins performed in industry and thus function for an increased number of cycles which is of notable economic importance. However it is to be noted that small molecule ligands make for less specificity, leading to copurification of other factors and low productivity of the process, which is a key disadvantage for their routine use. Although affinity chromatography can be a very useful method in terms of selectivity and affinity, it is never used independently for the purification of recombinant proteins, mainly due to the fact that access to suitable ligands is often scarce and endotoxin and glycoform contamination has also to be taken into account.

### 2.2. Ion-Exchange Chromatography

Ion-exchange chromatography (IEX) still remains one of the most frequently implemented initial steps for protein separation in industry and for most of academia, provided that the target protein is not tagged and not an mAb. There are many reasons for this, such as the fact that proteins are amphoteric molecules and any protein will bind to an ion-exchange resin depending on the pH of the solution. IEX provides high resolution under mild conditions with high binding capacity. The pH of the buffer selected for binding and elution affects the charge on weak ion exchangers but not on strong ion exchangers which retain their charge over a wide pH range. Hence, they are usually the resins of choice in commercial bioprocess operations. Pilot-scale studies are usually carried out to determine the binding of a target protein to a resin of interest and to determine the effect of different ionic concentrations and pH before scaling-up. Elution is performed by altering the pH or ionic strength of the solution and because the reproducibility of the pH shift is less, a salt gradient is preferred on a large scale. Theoretically, any salt can be used for elution because all of them modulate electrostatic interactions and thusbinding and elution.

The “salting-out” effect of a salt on a protein, its valency, and ion binding to proteins determine the elution properties of a salt, which explains the different elution properties of salts with the same valencies [55]. “Salting-out” can induce higher binding to IEX resin and therefore salts such as sodium chloride (NaCl) which have weak “salting-out” effects on proteins are preferentially selected for elution over high “salting-out” salts such as ammonium sulphate (NH_4_)_2_SO_4_. Ion exchange can be robustly used for aggregate removal as shown in studies for the mAb and other recombinant protein purification [56, 57]. Moreover, the presence of 0.15–0.2 M arginine in the binding buffer was shown to be more effective than salts like citrate and NaCl in reducing aggregate formation during elution [58]. Even when the resin that binds the target protein is a cation exchange (CEX) resin, an anion-exchange (AEX) resin is often used in tandem for effecting the removal of deoxyribonucleic acid (DNA) and lipopolysaccharide (LPS) impurities from the feed [59]. Usually, IEX and other chromatographic methods require the feed (or loading material) to be free from cells and cellular debris, which introduces an additional centrifugation or filtration which may be costly and/or time consuming.

The removal of low-level impurities structurally related to the product has been a challenge for the bioprocess industry and traditionally this has been facilitated by using shallow linear salt gradients at low column loadings, hence limiting the throughput [60]. Displacement chromatography incorporates an element which displaces the components of loading feed by having a higher affinity for the stationary phase which results in a zone of purified feed components that runs ahead of the displacer. Different types of displacers may also bind to resin-bound protein and enhance the binding, resulting in the displacement of the protein to the displacement zone.

Ion-exchange displacement chromatography has received attention for being a powerful technique, facilitating the purification of biomolecules, and one of the reasons for this relates to its superior resolving power in differentiating between related protein variants [60]. Another demonstration of its high resolution is the fact that ion-exchange displacement chromatography can separate proteins having similar retention properties in traditional IEX [61]. In addition, there is a wide variety of choice available for selecting suitable displacers, and these include carbohydrates, their derivatives, proteins, and even low molecular mass displacers like dendrimers, protected amino acids, and antibiotics [62–66]. Furthermore, high-throughput screening can be employed to screen a library of displacer analogues to select the most applicable displacer for purification of target protein present in a mixture of proteins [61]. However removal of displacers from target molecule solutions and their recycling issues, leading to increased costs, are disadvantages of this mode of chromatography. Moreover, because displacers have high affinity for the resins, their removal from column during regeneration presents a challenge and extreme pH or very high percentage of organic solvent may be necessary. This reduces throughput and increases costs. Another interesting study describes an alternative mode of IEX, namely, back-flush ion exchange chromatography which was implemented for purifying recombinant aldehyde dehydrogenases (ALD4p and ALD6p) and a methyltransferase (Bud23p) from *E. coli* extracts in a single step. In this method, sample was loaded onto a Q sepharose Fast Flow column in the opposite direction to which it is eluted, and the pH of the sample was kept higher than the pI value of the target protein [67]. Keeping the pH high kept impurities held back to be eluted in high salt concentrations and protein of interest was eluted as a single peak with little or no protein eluting with it. However, the binding conditions for this technique has to be empirically determined for every protein in question as other target proteins in the study did not bind at the pH which was used for Bud23p.

### 2.3. Hydrophobic Interaction Chromatography

Hydrophobic interaction chromatography (HIC) is a popular method for protein purification, and, similar to other chromatographic techniques, the ability to integrate mathematical modelling of the process and to calculate the hydrophobicity of the target protein simplifies the design of purification processes. Proteins bind to HIC hydrophobic ligands on resins based on their hydrophobicity, which is also indicative of the separation conditions such as retention times of a given protein and therefore performance of HIC [68]. Protein hydrophobicity is determined by the amino acids that constitute the proteins, with high concentrations of salt (e.g., ammonium sulphate) exposing the surface hydrophobic patches and thus enabling the binding of proteins to the HIC column. Subsequent elution is performed by a decreasing gradient of salts. This makes HIC a method of choice just after IEX-based elution or ammonium sulphate precipitation during a purification scheme, as eluate and precipitate have high salt concentrations which favours binding to HIC resins. Theoretical methods to determine a target protein's hydrophobicity so as to predict the retention of protein on a given HIC resin have been described previously [69]. Furthermore, amino acids (glycine and arginine), polyols (ethylene glycol and glycerol), and sugars (sucrose) have also been shown to modulate binding and elution of proteins to HIC resins [70]. The use of “salting-out” salts results in low protein recovery and the application of stronger conditions present the risk of denaturing protein during the purification. Solvent modulators can be used to overcome this problem. For example, arginine, up to a certain critical concentration, has been shown to weaken the protein binding to HIC resins [71] and has been implemented for the elution of interleukin-6 (IL-6) and monoclonal antibodies [58]. In some cases, the effect of arginine is more dramatic, for example, for activin [71]. To elaborate, as a sticky protein, activin was not eluted from the HIC resin by low citrate concentration and even by 20% (v/v) ethanol. However, 0.5 M arginine resulted in 60% recovery of this protein, with a sharp elution peak of activin observed [71]. In summary, HIC can be used for separation of recombinant proteins, homologous proteins, and antibodies and since the separation of protein on HIC operates on a different principle to IEX and other purification methods, it can be applied in conjunction with these methodologies to purify proteins from very complex biological mixtures [72]. Moreover HIC operates on high-salt binding conditions and IEX elutes in them; it can be used as a next step after IEX without the requirement of a buffer exchange.

### 2.4. Mixed-Mode Chromatography

Mixed-mode or multimodal chromatography has also recently being developed and applied to a wide range of purification processes [73]. The lack of requirement for high ionic strength for solute binding to mixed-mode resins, facile elution and the unique selectivity offered by these resins is enhancing their popularity among the industrial community for protein purification schemes. Principles, characteristics and design of ligands for mixed mode chromatography have been discussed previously [74]. Mixed-mode resin selectivity results from electrostatic interactions and hydrophobic interactions as well as hydrogen bonding, depending on case-to-case basis. Hydroxyapatite (HA) chromatography is a traditional example of mixed-mode chromatography in which protein can bind to resin via either HA-phosphoryl (cation-exchange) or HA-calcium residues (metal affinity). Small basic proteins usually bind to the HA by phosphoryl-cation exchange and acidic proteins interacting predominantly by calcium affinity. However, large proteins can bind using both mechanisms [75]. HA is available in either microcrystalline form or as spherical particles. Microcrystalline HA is not mechanically stable, which limits its use in batch mode only, while spherical particles can be used over multiple cycles [76].

A popular HA used for recombinant protein purification (e.g., antibodies) is ceramic HA (CHT by Bio-Rad Laboratories). HA chromatography is also particularly useful for removing aggregates of proteins which may be present alongside the target protein in a crude mixture. Furthermore, the presence of poly-ethylene glycol (PEG) in the binding buffer enhances the retention of protein on the HA column, and this is proportional to the size of the target protein. This results in aggregates of the protein being retained on the column for longer than their monomeric counterparts, thus facilitating the removal of aggregates [75]. A clear separation of leached protein-A from the previous chromatography step, DNA, endotoxins, viruses, host cell proteins, and aggregates from monomeric immunoglobulin G (IgG) has been reported using CHT [77], which further demonstrates its efficacy. In a further application of CHT, mAb specific for Japanese encephalitis virus was purified in two steps using HA chromatography and membrane filtration without using the usual protein-A step [78]. In this study, 90.2% purity along with 90% recovery was achieved and antigen binding activity was preserved in the product. The running time was reduced to one-third of the conventional method which would reduce operation cost in a process. However, mechanical instability, low reusability, and high costs are some of disadvantages of CHT chromatography.

Other mixed-mode resins have also been developed and a number of companies have brought their own mixed-mode resins to the market. Some of the commercially available multimodal media and their characteristics are outlined in Table 2. In one study evaluating mixed-mode resins, to remove aggregates from antibody preparations, Capto adhere (GE healthcare, Piscataway, NJ, USA) and Benzylamine (BA, prepared according to [79]) were able to remove aggregates from 20.5% to 2.6% and 2.4%, respectively, for a load of 50 mg/mL of resin [80]. In the same study, the ability to remove aggregate from antibody preparations by resins employing electrostatic interactions (Q Sepharose FF) and hydrophobic interaction (Phenyl Sepharose 6 FF) was found to be lower compared to mixed-mode resins (Capto adhere and BA) employing both types of interactions. In another study, Capto adhere, for loading up to 100 mg/mL of resin, was able to reduce aggregates from 10.5% to 2.3% while traditional anion-exchange resins showed a reduction from 12.8% to 11.4% [81]. However IEX is a good technique to reduce aggregates but mixed-mode chromatography demonstrably performs better than IEX in aggregate removal and should be tested in larger studies with more varied type of culture mediums. As more and more resins become available with unique selectivity and higher binding capacities, this will likely result in mixed-mode chromatography becoming more frequently used in future academic and industrial protein purification applications.

### 2.5. Size Exclusion Chromatography

Size exclusion chromatography (SEC), also referred to as gel permeation chromatography or molecular sieving, separates molecules based on their hydrodynamic volume and has been implemented for several decades for protein purification applications. Here, retention times for larger molecules are shorter than for smaller analytes and hence this property permits efficient separation, provided that assay conditions are optimised. The same principle can be taken advantage of if trying to desalt or exchange the buffer of a protein solution for another buffer. Resolution between differently sized molecules is a function of resin pore size, bed height, flow rate, mobile phase composition, and sample size. SEC stationary phases typically have a low resolution in separating molecules of different size. Therefore, the careful selection of a suitable stationary phase is an absolute necessity, especially, considering the size of the target molecule and associated superfluous contaminants. The retention time of a protein and its subsequent resolution are affected by the composition of the mobile phase. Proteins interact with stationary phase molecules by electrostatic and hydrophobic interactions which delay their retention times, resulting in a loss of resolution or increased peak volume which might not be desirable [82]. A decrease in mobile phase pH lowers the ionisation of acidic proteins. Furthermore, low ionisation reduces the stationary phase-protein interaction of acidic proteins. The pH of the mobile phase can be selected taking into consideration the nature of the target protein to be isolated and contaminants, so as to increase the retention time of the contaminant(s) and reduce that for the target protein, or *vice-versa*. Another factor influencing the retention time is ionic strength. High phosphate concentrations and moderate NaCl concentrations suppress the electrostatic interaction of proteins with the stationary phase [83]. However, high concentrations of, for example, ammonium sulphate may enhance the protein adsorption to the stationary phase through hydrophobic interactions which increases retention. The inclusion of organic solvents such as acetonitrile in the mobile phase can reduce these interactions [84] but may also denature the target protein and result in the underestimation of aggregate content. However, the use of 0.2 M arginine was shown to reduce protein-stationary phase interactions, increasing recovery of a mouse mAb by 2.39-fold and improving the accuracy of aggregate content analysis when recombinant human activin, interleukin-6, basic fibroblast growth factor, mouse mAb (purified from myeloma cell conditioned media), and interferon-*γ* were used as a mixture of model proteins [85].

Proteomic approaches for selecting a purification method based on physicochemical properties, and optimisation of operating conditions using mathematical modelling have been described previously [86]. Because of its poor resolution and excellent desalting properties SEC is mostly used as a polishing step when the volume has been reduced and the removal of aggregates and a change of solution are necessary. Moreover, samples volumes needed for SEC are very small therefore a concentrating step is needed increasing the costs of the process. To achieve good separation in SEC, long columns are needed and low flow rates are necessary due to back pressure constraints and both of these factors reduce the productivity. These above-mentioned issues are key disadvantages of SEC in industrial settings. High-performance tangential flow filtration can also perform concentration and buffer exchange in a single step (Section 4) and this methodology appears to be gradually replacing SEC in industrial downstream processing. However, SEC is widely used as an analytic technique to estimate and monitor percentage of aggregates in a given sample.

## 3. Continuous Chromatography

Typically in traditional column chromatography, for example, protein-A chromatography, the column is loaded up to 90% of 1% breakthrough capacity which leads to insufficient utilisation of the column's capacity [87]. Moreover, while the end of the column remains unsaturated, the entrance to the column is saturated resulting in excess buffer consumption used in washing and elution stages [88]. These factors limit throughput at preparative scale. Notwithstanding these limitations, some of these factors can be overcome in a continuous operation where continuous product injection and product withdrawal are performed. There are multiple types of continuous chromatography models and operations. For example, multicolumn counter current solvent gradient purification is a process capable of providing high yield and purity of products [89, 90]. It is particularly useful for resolving variants of proteins with an intermediate product being accompanied by weakly and strongly adsorbing variants such as mAb variants [91, 92]. It has, however, been successfully applied to purification of mAbs directly from cell culture supernatant as well [93].

The major advantages of the counter current-simulated moving bed technique are reduced solvent consumption, increased productivity, and more concentrated product which collectively contribute to cost reduction in any given process [94]. These continuous operations have been recently used successfully for purification of recombinant streptokinase [95], mAb [87, 88], single-chain antibody fragment [31], and capture of IgG on a cation-exchange column, [96]. Of notable interest is the observation that each of these studies reported either significant cost savings and/or better productivity and performance compared to batch mode chromatography. In recent years, continuous chromatography has been employed in a variety of chromatographic systems and applications including protein-A affinity [87, 88], cation-exchange [96], immobilized metal ion affinity chromatography [31], and hydrophobic interaction chromatography [95]. 

One major attraction of using counter current chromatography is predictable scale-up of the process from test tube analytical scale to pilot/process scale. For example, a scale-up from 5.4 mL to 4.6 L using the calculations done on test tube scale (calculation of distribution ratios) have been reported by Sutherland et al. [97]. The retention time for binary separations (benzyl alcohol {4.5 min} and p-cresol {6.5 min}) was similar however; resolution fell down from 1.54 to 1.01 [97]. However, the separation of the binary mixture was not significantly affected. Continuous separation in a protein production plant is very attractive in terms of cost as well. It was shown in a study that variable costs for operations of continuous systems are approximately 4.0 M $/year lower than that of batch separation systems [98]. This is because of the lower operating costs (resin efficiency, less solvent consumption, etc.) of the continuous plant.

## 4. Field-Assisted and Electrophoretic Separation Methods

**Figure 1:**
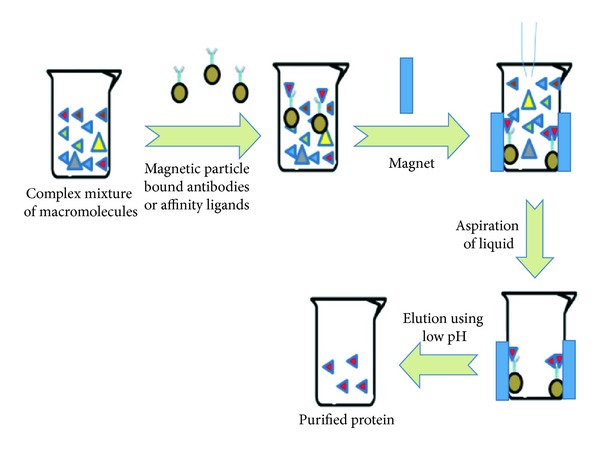
Magnetic separation of macromolecules from a complex sample matrix.

As mentioned previously, downstream processes account for the majority of the cost during the production of a biopharmaceutical preparation and, hence, there is a constant need for the development of novel, improved, and more cost-effective methods for protein separation. Electric, acoustic and magnetic fields can be used alone or in conjunction with established separation methods (e.g., membrane-based methods) for the separation of proteins. With reference to the latter, bio-macromolecules such as proteins and cells exhibit ferromagnetism, paramagnetism, and diamagnetism [99]. Although magnetic separation of cells is out of the scope of this review, fermentation media may be frequently contaminated with cells which need to be separated from the protein of interest. Magnetic particles bearing affinity ligands for cells may be added to the medium and separated by a magnet, a methodology which has routinely been used for the isolation of microbial cells from culture medium [99]. Affinity-based magnetic methods similar to that seen for the separation of cells have also been used for separating proteins, DNA and ribonucleic acid (RNA) from nutrient media, fermentation broths, tissue extracts and biological fluids [99]. A schematic representation of the process involved in the magnetic separation of macromolecules or cells is shown in Figure 1.

Another field-assisted technique, namely, free-flow electrophoresis (FFE), may be implemented for the purification of cells and proteins from different sources, including human sample matrices (e.g., urine and serum). FFE has some advantages over chromatographic methods such as continuous sample injection and the ability to separate crude extracts containing cells and subcell particles. On the other hand, some disadvantages for scale-up are also evident, such as the need for specialised instrumentation and the requirement for the optimisation of multiple parameters for complex sample analysis. FFE was used for IgG purification by human plasma fractionation in a single step and 79% recovery with clinical grade purity (IVIG grade) was achieved [100]. In the same study, when process was adjusted to become two-phase process, 93% recovery and 98.8% purity were achieved and the process could be scaled up to 100 times with 93% yield of IgG. In another study, the complete purification of cytochrome C and myoglobin from a binary mixture was achieved by a combination of FFE and a micromodule fraction separator [101]. Recombinant human growth hormone (rhGH) was purified (98%, with 90% yield) from culture medium of CHO cells in a single step using gradiflow preparative electrophoresis and a 50 kDa separation membrane, which demonstrates the power of electrophoresis in separating proteins [102]. Gradiflow preparative electrophoresis is easily scalable [103] and can be used for the purification of recombinant proteins at large volumes. In a separate study, FFE was compared with multiple chromatographic and filtration steps for permitting the purification of mutant recombinant tumour necrosis factor-*α* (TNF-*α*) and the yields and purity were comparable for both procedures [104]. Finally, preparative electrophoresis is a powerful technique for the purification of protein and although it has been utilised to a lesser extent during large-scale purification of proteins at industrial and academic levels, it warrants further attention due to its high resolution and ability to perform continuous separations in a cost-effective manner by replacing multiple chromatographic steps. However, issues in scale-up and complex method setup have to be taken into account, and, theoretically, continuous chromatography would fare better in an industrial process.

## 5. Membrane-Based Systems

Membranes are interfaces acting as a barrier between liquid-liquid and liquid-vapour phase contacting two sides of an interface. Membranes are popular in the biotechnology industry for protein purification/concentration, depending on the size and/or charge of the target proteins. In addition, they offer key advantages pertinent to cost and ease of scaling-up over chromatographic methods. Currently, membrane-based systems are routinely used in industry and academia for downstream processing of recombinant proteins [105–107]. The highest interest among the use of membranes has been on the pressure-driven processes, such as ultrafiltration (UF), microfiltration, virus filtration, and nanofiltration. Ultrafiltration membranes are usually 1–20 nm in size and their protein retention is very high. They are mainly used for protein concentration and buffer exchange and appear to be used in preference to SEC on an industrial scale, [108]. The permeability of a membrane is determined by pore size distribution, porosity, thickness, and solvent properties. Any charge present on membranes will alter the flow and, consequently, protein transport across the membrane [109]. The hydrophobicity of the membrane also plays a key role and reduces the flow and process permeability due to protein adsorption [110]. Polyethersulfone (PES) membranes are more hydrophobic compared to their cellulose-based equivalents. Membrane fouling also has a similar effect on process permeability and is a limitation for the use of UF. Many modules like hollow fibre, spiral-wound, flat-sheet cassettes, and tubular modules have been developed for UF. They provide separation of retentate and filtrate streams, mechanical support, and easy access for cleaning and replacement, in addition to enhanced mass transfer properties [111].

Although UF is routinely used in industries for protein concentration and buffer exchange, protein separation by single step UF is still a challenge. However, there are several examples of protein purification from complex matrices using UF in the literature. Purification of recombinant collagen from corn extracts was achieved with up to 99% purity using UF membranes [112]. The permeate flux was improved with higher transmembrane pressure and high crossflow rates (.25 m/s). In another study, the purification of lysozyme from chicken egg white and the associated effect of different parameters, such as pH, system hydrodynamics, feed concentration, and transmembrane pressure on permeate flux, have been studied using hollow-fibre PES membranes [113]. Here, it was observed that system hydrodynamics did not affect the purity or lysozyme transmission through the membrane, whereas higher retention was observed at higher crossflow velocities due to increased permeate flux. Three-or four-stage cascade UF was employed for lysozyme purification from chicken egg white and in four-stage cascade 97% purity and 71% recovery were achieved using PES and polysulfone membranes in flat-sheet tangential flow and hollow fiber mode [114]. Negligible membrane fouling was found in this system and internal flow rates affected product recovery and not purity. UF membranes can also be made of polyacrylonitrile, cellulose, and cellulose acetate and ceramic, among other materials. Traditional UF membranes mostly differentiate between proteins based on their respective sizes, and a tenfold difference is usually necessary for effective separation. However, charged membranes have shown more promise in permitting the separation of proteins like myoglobin, bovine serum albumin (BSA) and recombinant fragment antigen binding (Fab) fragment and a full-length antibody [115, 116].

Ultrafiltration under the effect of an electric field (electro-ultrafiltration, EUF) is also being analysed for protein purification and, in one study, it increased flux permeate by 25–50% in a protein solution of 1–5 g/L compared to the UF performed in the absence of any electric field [117]. EUF has also been used to reduce membrane fouling and applying a critical voltage eliminates particle deposition at the membrane and restores the flux [118, 119]. It was identified in one study that the most suitable conditions resulting in the highest average filtration flux can be reached by applying higher voltage, shorter pulse intervals, and longer pulse durations [120]. However, electrolysis occurring at the membrane and resulting local pH changes might result in inactivation or denaturation of target molecule. The application of ultrasonic energy at high power to a liquid produces acoustic streaming and shear forces by cavitations in a liquid, which leads to reduced membrane fouling and increased permeate flux [121]. Application of higher frequency ultrasound leads to greater acoustic streaming flow rates compared to lower frequencies for the constant power intensity [122]. In some studies it was found that electric and ultrasonic fields had synergistic effects when both fields were applied simultaneously [123, 124]. Electric field assisted separation for lysozyme and bovine serum albumin has been reported [125]. Recently, using the same technique, purification of soy peptides from a complex mixture was achieved [126]. However, the modelling of retention under the effect of electric field is complex and this might delay the development of this technique for recombinant protein purification.

**Figure 2:**
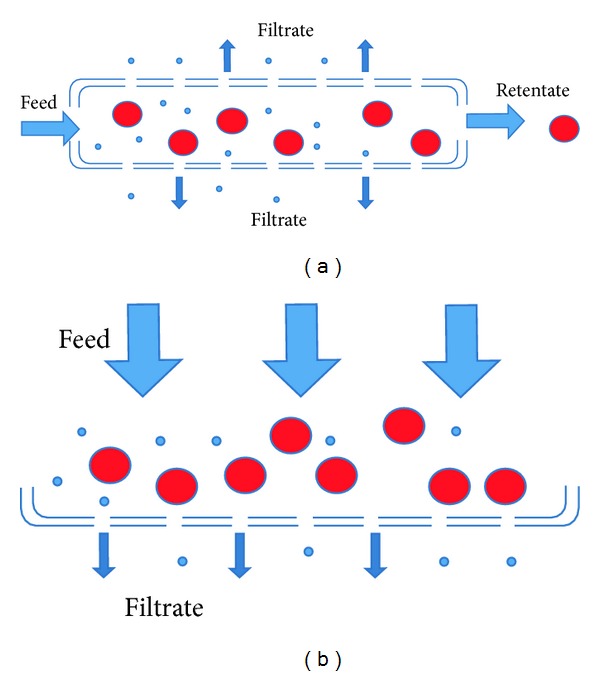
Tangential flow (a) and dead end filtration (b) methodologies.

Another emerging technology in membrane separation processes is high-performance tangential flow filtration (HPTFF). A schematic representation of dead-end filtration and tangential flow filtration is shown in Figure 2. Conventional tangential flow filtration can separate molecules differing in size up to 10-fold or higher, but HPTFF can distinguish between molecules differing in size by up to 3-fold by optimisation of buffer and fluid dynamics and through the use of selectively charged membranes. HPTFF takes into account both the size and charge of a molecule and can perform protein concentration, purification, and buffer exchange in a single operation, hence reducing the cost of downstream processing [127]. At their isoelectric points, proteins have a net neutral charge and, therefore, have no ionic layer and a low effective volume which results in a high sieving coefficient and high passage of protein through the membrane pores. This effect is more pronounced at low ionic strength concentrations, and high ionic strength reduces the effective volume which in turn changes the sieving coefficient. Hence, careful optimisation of buffer pH, ionic strength and, the use of charged membranes result in a process which is highly selective for product protein retention and allows impurities to pass through [128]. HPTFF has been used to separate protein monomers from oligomers, protein variants differing in only one amino acid, and a Fab fragment from a similarly sized impurity which demonstrates the high selectivity which can be obtained [129, 130]. More recently, this technique, in combination with anion-exchange membrane chromatography, has been used to purify recombinant penicillin acylase from bacterial cultures with relatively high purity 19 units/mg and recovery (72%) [107]. Clarification of the culture was simultaneously achieved making the process economical requiring less steps.

An alternative membrane-based separation process, membrane chromatography, uses microfiltration or larger pore size membranes which have ligands immobilised to the inner pore surface which results in the highly selective separation of proteins. Ion-exchange, affinity reversed phase, and hydrophobic interaction membranes have been developed and Pall (New York, USA) Mustang Q and S membranes are examples of anion- and cation-exchange membranes, respectively. Flat sheet, stacked sheet, and pleated modules are also available for membrane chromatography applications. Membrane chromatography has been applied in both flow through and bind-and-elute industrial applications [111]. For flow through applications, process parameters are optimised such that impurities are bound and retained allowing the product of interest to flow through. For example, in one study leached protein-A and aggregates of mAb bound reversibly to the poylvinylidene membranes while mAb flowed through separating it from impurities [131]. This application was based on hydrophobic interactions and membrane was regenerated by lowering the salt concentration releasing the aggregates and protein-A. This methodology has also been used in bind-and-elute applications for DNA, RNA, and viruses [132, 133]. In terms of dynamic binding capacity and economics, membrane chromatography fares well with beads-based media for larger solutes (such as mAbs) but cannot compete for smaller solutes (smaller proteins). However, developments in membrane structure and device design should compensate for this in future applications [111].

## 6. Phase Partitioning

**Figure 3:**
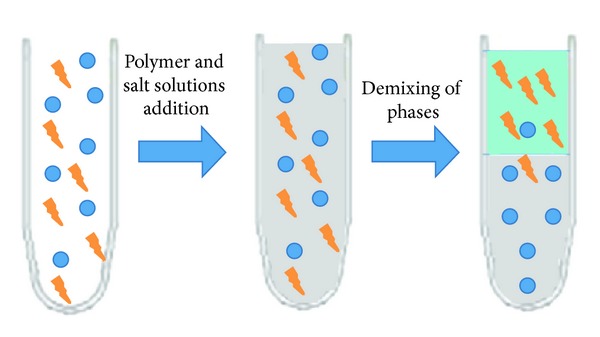
Separation of constituents through the implementation of aqueous two-phase partitioning.

Out of the all the downstream processing steps, the selective purification steps represent 70% of the total costs and are dominated by chromatography-based methodologies such as those outlined above [134]. Notably, the increasing upstream titres required to satisfy the demands for higher protein yields place considerable economic and physical pressure on these systems. This means that there is an absolute requirement for the development and implementation of alternative steps which either purify the proteins independently or reduce the burden on the normally applied purification step (e.g., chromatography) by partial purification. One of these systems is liquid-liquid extraction, for example, aqueous two-phase partitioning. Aqueous two-phase systems are formed by mixing of two aqueous solutions of structurally different components above a certain critical concentration [135]. These different components can be two different polymers or a polymer and a salt (Figure 3). In downstream processing of proteins, product phase consists of water, and most polymers have stabilising effects on protein structure [136]. Therefore, these techniques are compatible with protein-producing systems and offer the advantage of combining concentration and purification in a single step with easy scale-up.

Aqueous two-phase extraction (APTE) has also been used to purify cells, viral particles, and plasmid DNA [137–139]. Properties of biomolecules, like hydrophobicity, surface charge, size, and system composition, affect the partitioning of the molecule in any two-phase system. This implies that partitioning of a protein in aqueous two-phase systems can be manipulated by altering the polymer mass, pH, ionic strength, and concentration of the phase component or through the addition of affinity ligands. This gives flexibility to design and allows for the optimisation of a system which targets the protein to one phase and most of the impurities to another. High-throughput screening can be used to determine the partitioning coefficient and effect of different phase component parameters like mass of polymer, pH, and ionic strength [140].

Factorial designs and central composite designs have also been used to screen and optimise the partitioning of a biomolecule and establish the effect of different parameters on partitioning [141, 142]. Traditionally polyethylene glycol (PEG)/dextran and PEG/salt (e.g., potassium phosphate) systems have been selected for use, but temperature-separating polymers, such as ethylene oxide-propylene oxide (EOPO), and pH-sensitive polymers for example, polydiallylamineethanoate-dimethyl sulfoxide (PAEDS), have also been reported [143–145]. After the biomolecule has been targeted to the polymer phase, temperature- or pH-sensitive polymers can be recovered by altering the pH or temperature to precipitate the polymer for further use, while the target biomolecule remains in the solution. Yields in separation by the EOPO system have been reported to be enhanced by the addition of nonionic detergents [146] or through the addition of a hydrophobic tag (tryptophan, tyrosine, or hydrophobin I) to the target protein [147]. Many reports discussing the APTE-based extraction of mAbs, insulin-like growth factor (IGF), hGH, and insulin have been reported in the literature [148].

Another system called three-phase portioning (TPP) has also been reported. Here, one phase is an organic liquid phase like t-butanol and another phase is a salt (e.g., ammonium sulphate), with an interphase spontaneously formed. TPP is particularly useful for the extraction of proteins from cell extracts, as cellular debris tends to partition to the organic phase, with nucleic acid partitioning to the interphase. Many important biomolecules such as green fluorescent protein (GFP), xylanase, and antigenic proteins have been recovered by using this methodology [149–151], demonstrating its feasibility to be integrated into a purification process. Recently, catalase was purified from sweet potato tubers in a single step using TPP with 14.1 fold purification [152]. However, recovery of organic solvent for multiple uses and validation of its successful removal from the final product are important considerations. APTE is becoming the method of choice increasingly in downstream operations of biopharmaceuticals because of the ease of scale-up, continuous operation, biocompatibility, low toxicity of polymers and chemicals used, and possibility of process integration [153–155]. Moreover, it can perform clarification, concentration and partial purification in one step but comparing it with other processes in terms of purity alone it does not fare well. Its ability to be integrated into any industrial process along with the above-mentioned examples makes this technique suitable for any purification process [156]. In case of TPP, the use of organic solvent might affect activity of some proteins and usability of TPP has to be determined on a case-per-case basis.

## 7. Endotoxin and Virus Removal

Endotoxin and pathogen removal from therapeutics of biological nature is of paramount importance primarily because of their human uses. Gram-negative bacteria are widely used for production of recombinant proteins for therapeutic uses which entails methods to reduce endotoxin levels to those permitted by regulatory authorities. The development of cost-effective and efficient methods for endotoxin removal is an ongoing challenge for the biopharmaceutical industry. Endotoxins are highly stable molecules resisting extreme temperature and pH which makes it challenging to just neutralise them using harsh conditions without the loss of activity of target recombinant proteins. Based on the unique molecular properties of endotoxins, many methods like affinity resins, membrane adsorbers, ion-exchange and hydrophobic interaction chromatography, two-phase extractions and ultrafiltration have been developed for endotoxin removal with varying degrees of success [157]. Most methods designed for endotoxin removal work on a case-to-case basis depending on the properties of target proteins, and a single broadly applicable method is hard to find. For example, positively charged proteins such as urokinase can be decontaminated using anion-exchange chromatography due to the net negative charge of endotoxin [158], but, negatively charged proteins will pose the problem of product loss because they will bind to the column with endotoxins as well. Likewise, small proteins can be ultrafiltered to remove large endotoxin aggregates but with large target proteins like full-length antibodies, this approach would not work due to overlapping in size of the product and impurity. Another major problem would be interaction of endotoxin with proteins which might affect their solution behavior and, consequently, the strategy to be implemented for effecting their removal [159]. Alcohols can be used to remove LPS from LPS-protein complexes although safer, non-flammable alternatives such as alkanediols have also been proposed and found to remove LPS in combination with ion-exchange chromatography [160]. More recently, various factors affecting the removal of endotoxin from protein solutions using anion-exchange chromatography were evaluated [161]. It was reported that pH could be kept sufficiently high to prevent the conferring of a positive charge to the protein to facilitate dissociation of the endotoxin bound to the protein. Thereafter, resin volume can be increased to prevent saturation of resin with endotoxin and some conductivity can be applied to maintain low interaction between endotoxin and protein. Keeping all these factors regulated, an endotoxin content of 0.5 EU/*μ*g and 80% product recovery were achieved. However, the success of this method may have something to do with the molecular properties of model proteins used in the study, although these factors can be considered while designing endotoxin removal methods for any target protein. Affinity resins including immobilised L-histidine, poly-L-lysine, poly (*γ*-methyl L-glutamate) and polymyxin B have also been traditionally used to remove endotoxins from protein solutions [162–165]. A new affinity ligand for endotoxin removal has been recently synthesised and was found to be better than histidine immobilised silica in removing endotoxins from BSA solutions [166]. Another interesting approach was implemented in which recombinant monoclonal antibody bound to an affinity resin was washed with 0.5 M arginine which resulted in a dramatic reduction of protein-bound endotoxin content and a final endotoxin content of 0.2 EU/mg of target protein with 95% product recovery was achieved [167]. This is an interesting method as arginine is a nontoxic amino acid and can be readily removed from protein solutions by washing the column if the protein is column-bound or by filtration if it is a protein solution. Another method for endotoxin removal which is very promising is aqueous two-phase micellar system (ATPMS). In ATPMS, an aqueous surfactant under appropriate solution conditions spontaneously separates into two phases, micelle-rich and micelle-poor phase [168]. A careful optimisation of the conditions can result in partitioning of target protein into one phase while the endotoxin partitions in another phase. Recently, one study described that green fluorescent protein (GFP) produced in *E. coli *was found to partition preferentially into micelle-poor phase, while LPS partitioned into the micelle-rich phase [169]. Previously, one study concluded that for decontamination of various proteins such as, troponin-I, myoglobin, and creatine kinase, a two-phase extraction using Triton X-114 was noted to provide improved performance relative to affinity adsorption [170]. A separate study noted that a polymyxin B resin was found to be to equivalent to APTMS in removing LPS from DNA preparations [171]. Therefore, when referencing the literature, one does not find a single method which is broadly applicable to remove endotoxin from any target protein and a case-to-case method development and validation are a recommended strategy to implement.

The strategies used for virus removals from recombinant proteins including antibodies are low pH inactivation, detergent treatment, membrane filtration, protein-A chromatography, and AEX [172, 173]. One important consideration in protein-A and AEX based methods is the robustness of the virus removal over recycling of the resin. Protein-A chromatography removes viruses from antibody solutions by allowing them to flow through the column while retaining antibodies [25]. The robustness of the clearance is dependent on the lifetime of the column and can be reflected in step yield and antibody breakthrough (concentration of antibody passing in effluent) [174]. In the case of AEX, the robustness can be reflected in band spreading, an increase in back pressure, or impurities in process fluid [175]. These factors should be checked to determine the robustness of the process in virus removals. Since, AEX shows DNA binding, it is superior in removing viruses from the feed. In virus filtration, membrane fouling over time can reduce the effectiveness of virus clearance probably owing to reduced pore size [176]. Emerging technologies for virus removal are smartly designed ion-exchange or ligand-coupled membranes which are disposable [177]. One clear advantage of this method is that one can design very tight-binding membranes because the binding does not need to be reversible. These membranes allow very high flow rate and therefore short processing time and thus are cost effective and validation becomes easy because of them being disposable.

## 8. Future Trends and Conclusion

The purification of expressed protein is usually the bottleneck in the cost of protein production and this has put immense pressure on the need to develop novel purification methods and make improvements to existing strategies. Here, we have discussed the rapid development of affinity chromatography and how combinatorial approaches for ligand discovery are revolutionising this field. These ligands can be engineered to be made stable at the wide pH and temperature ranges used in cleaning and sterilisation of affinity columns, thus simplifying their use over multiple purification cycles. This ultimately reduces the cost of the purification process while also increasing the product yield. Due to its high selectivity, affinity chromatography is usually the preferred first step in purification of recombinant proteins on small or larger scales. The use of protein-A chromatography for antibody purification, notwithstanding its high costs, is an example. It should be noted that proteinaceous ligands for use in affinity chromatography applications have their limitations as they cannot withstand the harsh cleaning conditions introduced between purification cycle batches, which limits their reuse and increase associated costs. An improved protein-A ligand engineered to withstand cleaning with 0.5 M NaOH has been reported, and there is notable interest in its use in industrial downstream processing for purifying recombinant antibodies.

Separately, the use of combinatorial chemistry and phage-display technologies is generating a large repertoire of ligands specific for virtually any protein. It is likely that these ligands will be increasingly used in future applications for large-scale recombinant protein purification. Companies like SomaLogic (CO, USA) and VersaMatrix (Denmark) have launched custom ligand discovery services based on these approaches, and future applications comprised of reduced numbers of purification steps look increasingly promising. Furthermore, existing chromatographic processes are constantly improving and processes such as back-flush ion exchange and displacement chromatography are continuously in development. Once these and other methodologies are fully developed, this will lead to significant improvements in selective purification of target proteins.

The limiting factor for large-scale use of displacement chromatography is the lack of selective displacers. However, many of these have recently become commercially available, suggesting that this technology will become increasingly more popular for industrial purification processes. Many companies have developed mixed-mode stationary phases which offer unique selectivity and higher capacity than existing media. These media for IEX, HIC those of and mixed-mode chromatography are also more robust and can tolerate the sanitisation conditions which consequently allow multiple reuse. In case of processes where affinity ligands are not costeffective and traditional ion exchangers are unsuitable, mixed-mode media can provide an alternative which reduces the number of process steps and provides unique selectivity. An increasing number of companies are launching mixed-mode media into the market which are robust for in-place cleaning and provide unique selectivity for binding to either the target protein or impurities which reduce the cost of the process by lowering the number of process steps, which is a key economic consideration. Trend will be towards continuous processes for all types of chromtographies as this required less buffer consumption and results in high productivity compared to batch processes.

Large bead hydrogel-based technologies such as inside-out ligand attachment (IOLA), developed by LigoChem (NY, USA) are also very promising. IOLA promotes ligand attachment to the inner surface of the beads which are chosen based on target protein size, and larger impurities including viruses and other proteins cannot enter the beads and, thus, are washed out. In addition, HPTFF and membrane chromatography are rapidly emerging systems for protein purification and provide alternatives to traditional chromatographic systems. Although currently membrane chromatography cannot compete with traditional bead-based chromatography for binding of small solutes, newer developments should address this drawback. Membrane chromatography is already popular for viral removal, and this technique has immense further potential. For example, membranes with protein-A mimetics or other affinity ligands for target proteins can be envisioned in future developments. We also postulate that, by making improvements to the flow characteristics of membranes, this will result in enhanced performance and lead to higher protein purification titres.

We have discussed how aqueous two-phase partitioning is very selective, user friendly, and rapid. Notwithstanding their high costs, associated polymers are nontoxic, which is an important benefit for processes seeking approval for the production of therapeutics intended for human use. However, it should be noted that an important drawback of phase partitioning separation methods for industrial uses is the slow rate of phase demixing. However, field-assisted demixing and electroextraction appear to overcome this limitation. ATPE is also attractive because it can concentrate the target protein with a high degree of separation during the initial stages of the purification, which ultimately reduces the burden from subsequent chromatography-based steps. It should also be acknowledged that a limitation of ATPE which requires further studies is the scarcity of knowledge pertinent to the mechanism and partitioning behaviour of different proteins in various ATPE systems. This makes mathematical simulation of ATPE process difficult as it is not possible to accurately predict the partitioning of proteins.

Endotoxin removal is an important consideration in any pharmaceutical of biological origin produced in bacteria. Although detergents are effective in reducing endotoxin content during chromatography, on a larger scale, they would be very costly. Alkanediol and alcohol are alternatives and alkanediols should be preferentially selected owing to safety. One interesting and working approach would be arginine washing coupled to affinity chromatography which has been shown to work well. APTMS is a new and very promising technique which can be implemented in early stages of downstream processing to remove the majority of endotoxins. However, process parameters for Triton X-114 are not well defined and future studies are needed. Moreover, new chemicals which break endotoxin-protein complexes without affecting protein binding to chromatographic resins or endotoxin binding to affinity resin could also be a viable strategy. While, in case of virus removal, membranes with ion-exchange or affinity functionality seem to be the future trend with associated advantage of nonreversible binding and ease of validation.

Finally, it should be concluded that the efficient and cost-effective purification of proteins from a complex biological mixture is an important challenge for industrialists and academics alike, and currently none of the aforementioned methods can be used independently for efficient protein purification. However, mathematical modelling (simulation) and high-throughput screening of operational conditions in downstream processing have become integral parts of these processes and will play an even greater role in future applications for the design of product-specific downstream strategies. Furthermore, as more innovative technologies are developed and demonstrated to be effective for purification applications (e.g., single-use or disposable purification platforms), it appears likely that these will be applied more frequently in biopharmaceutical processes for lowering costs, reducing analytical times, and increasing yields of target proteins.

## Figures and Tables

**Table 1:** A panel of commonly used affinity tags selected for purification of recombinant fusion proteins and their associated characteristics.

Tag	Size [amino acids or kDa]	Ligand or separation method	Reference^a^
Polyhistidine	5–15 a.a.	IMAC	[6]
HA-tag	9 a.a.	mAb based	[7]
FLAG	8 a.a.	mAb based	[8]
Strep tag I	9 a.a.	Streptavidin	[9]
Strep tag II	8 a.a.	Streptactin	[10]
Softag 1	13 a.a.	mAb based	[11]
Softag 3	8 a.a.	mAb based	[12]
T7-tag	11–16	mAb based	[13]
c-myc	10 a.a.	mAb based	[14]
S-peptide	15 a.a.	S-protein	[15]
Polyaspartic acid	5–16 a.a.	Ion-exchange or precipitation	[16]
VSV tag	11 a.a.	mAb based	[17]
Calmodulin binding peptide	26 a.a.	Calmodulin	[18]
Glutathione S-transferase	26 kDa	Glutathione	[19]
Maltose binding domain	40 kDa	Maltose, amylose	[20]
PinPoint (Promega)	13 kDa	Streptavidin/avidin	[21]
Cellulose binding domain (Novagen)	27–189 a.a.	Cellulose	[14]
Xylanase 10A	163 a.a.	Cellulose	[22]

^a^Only one relevant reference is given.

**Table 2:** Selected commercially available mix-mode media.

Media	Supplier	Type	Ligand	pH stability
CHT ceramic hydroxyapatite	Bio-Rad laboratories	Ion exchange, metal chelation	[Ca_5_[PO_4_]_3_OH]_2_	Operating pH: 5.5–14 Can be cleaned with 1-2 M NaOH
CHT Fluorapatite	Bio-Rad laboratories	Ion exchange, metal chelation	[Ca_10_[PO_4_]_6_F]_2_	Operating pH: 5–14 Can be cleaned with 1-2 M NaOH
MEP	Pall life sciences	Hydrophobic binding near neutral pH, elution by pH reduction	4-Mercapto ethyl pyridine	Working pH: 2–12 Cleaning pH: 2–14
HEA	Pall life sciences	Hydrophobic binding near neutral pH, elution by pH reduction	Hexylamino	Working pH: 2–12 Cleaning pH: 1–14
PPA	Pall life sciences	Hydrophobic binding near neutral pH, elution by pH reduction	Phenylpropylamino	Working pH: 2–12 Cleaning pH: 1–14
MBI	Pall life sciences	Hydrophobic binding at acidic pH, elution by raising the pH	2-Mercapto-5-benzimidazole sulfonic acid	—
Capto MMC	GE Healthcare	Multimodal cation exchange	2-Benzamido-4-mercaptobutanoic acid	Long term: 2–12 Short term: 2–14
Capto adhere	GE Healthcare	Multimodal strong anion exchange	N-benzyl-N-methyl ethanolamine	Long term: 3–12 Short term: 2–14
